# External validation of a clinical decision rule: we need events in a population in order to rule them out!

**DOI:** 10.1186/s13049-016-0346-9

**Published:** 2017-01-13

**Authors:** Yonathan Freund, Anne-Laure Philippon

**Affiliations:** 1 Emergency Department, hôpital Pitié-Salpêtrière, Paris, France; 2 Paris Sorbonne Université, Univ-Paris06, Paris, France

## Abstract

We respond to the Lorton et al. article on external validation of the PECARN study. With an event rate of only 0.6%, we believe that their study failed to confirm the safety of this rule. Such a low number of events should call for caution when interpreting the results of diagnostic tests.

To the editor,

Lorton et al. [1] recently published a prospective study that aimed to evaluate the diagnostic performances of the PECARN clinical decision rule (CDR). We congratulate the authors for performing an external validation of this rule, which aims to guide and reduce the number of computed tomography (CT) head scans among children with minor head trauma.

In their study, the authors reported a sensitivity of 100% (95% Confidence Interval [CI] 66.4 to 100.0%), with a wide 95% CI that they acknowledged would limit their conclusions. However, when relying on a CDR to decide whether or not a child should undergo a CT, clinicians need to know the false negative rate they are dealing with. Therefore, the negative predictive value (NPV) is of utmost importance in such studies. Accordingly, Lorton et al suggest that this CDR is safe because its reported NPV is 100% [99–100%]. Indeed, an NPV with the lower bound of the 95%CI higher than 99% seems almost perfect, and could be used to validate the safety of this rule.

However, we would like to highlight that a major limitation of these results is the very low rate of events (namely intracranial hemorrhage) in their study population. Only 9 children out of 1499 met the primary endpoint, a rate of 0.6%. Therefore, statistically, if the CDR used was a coin flip “heads or tails”, the NPV would still be 99.6% (95%CI of 99.0 to 99.9%).

Such a low number of events should call for caution in the interpretation of the results of diagnostic tests.
